# Difficulty in Writing Perceived by University Students: A Comparison of Inaccurate Writers with and without Diagnostic Certification

**DOI:** 10.3390/children8020088

**Published:** 2021-01-27

**Authors:** Chiara Malagoli, Mirella Zanobini, Carlo Chiorri, Lucia Bigozzi

**Affiliations:** 1 Department of Education, Languages, Intercultures, Literatures and Psychology (FORLIPSI), University of Florence, Via di San Salvi 12, 50135 Firenze, Italy; lucia.bigozzi@unifi.it; 2 Department of Education Sciences (DISFOR), University of Genoa, C.so A. Podestà 2, 16128 Genova, Italy; mirella.zanobini@unige.it (M.Z.); carlo.chiorri@unige.it (C.C.)

**Keywords:** learning disabilities, university students, writing skills, transparent orthography perceived difficulties

## Abstract

Research has shown that academic success is strongly associated with positive academic self-efficacy beliefs and that individuals with learning disabilities (LDs) usually report a lower perception of competence than their peers in most learning domains. The aim of this study was two-fold: (1) To compare the performance of inaccurate writers who were not diagnosed with an LD with that of students who were diagnosed with an LD, in order to identify which tasks were the most challenging for individuals with LDs, and (2) to investigate whether inaccurate writers with and without a diagnosis differ in terms of self-perceived difficulties. Two groups were selected from a total sample of 639 students attending seven Italian universities: The first group included 48 participants (24 females) with scores on writing tasks below the 5th percentile, and the second included 51 participants (24 females) who were diagnosed with an LD. The results showed that the two groups significantly differed in the articulatory suppression condition tasks, but not in the standard condition tasks. When groups were matched for performance on writing tasks, students who were diagnosed with an LD reported significantly more perceived difficulties than students without an LD. The implications of these results in terms of the self-efficacy beliefs of students with an LD are discussed.

## 1. Introduction

A nonnegligible proportion of students in mainstream higher education have learning difficulties [1]. Learning disabilities (LDs) are, in fact, a condition of the cognitive asset that is stable across time [2]; although this condition is permanent across an individual’s life span, the expression of these difficulties evolves and varies throughout development, so the literacy skills profile of a child with LDs can be different from the profile of an adult or a young adult with LDs. Therefore, investigating the evolution of LDs beyond school years appears fundamental for obtaining a broader understanding of development trajectories. Additionally, it is crucial to understand the specific difficulties that may emerge later, in adolescence and beyond. Whereas the literature is quite rich and broad with regard to the early development of LDs and the impact on learning in lower grades of education, e.g., [3,4,5,6], less is known about the possible persistence of specific difficulties in young adulthood, adulthood, and during the transition into higher levels of education. Specifically, regarding writing skills, to date, only a few studies have investigated the evolution of writing abilities in young adults specifically diagnosed with dyslexia, dysgraphia, and/or dysorthography in the past, prior to their entrance at university. Recent studies have outlined how students with writing disorders report more difficulties in specific learning-related tasks, e.g., [7,8]. This topic is particularly important to investigate since many academic assessments are made on the basis of written texts [9] and because poor spelling is likely to influence school grades, academic results, and possibly the general sense of efficacy of these students [10,11]. Indeed, because students with an LD are expected to achieve the same learning objectives as their peers, these difficulties may impact other important individual dimensions connected to self-efficacy, self-esteem, and self-worth.

### 1.1. Developmental Trajectories of Learning Disabilities (LDs) Later in Development

As mentioned above, LDs tend not to fade with the progression of learning, but the expression of the disorders can change over time. Depending on the resources and characteristics of individuals, it is fairly common for LDs to remain undetected until the beginning of secondary education. Genovese et al. [12] reported that 17% of students who contact diagnostic centers each year are secondary grade school students, and 92% of these students are confirmed to have a certifiable LD. It is often the case that an elevated IQ, a supportive context, a high level of motivation, a strong effort to pursue learning tasks, high adaptive skills, or other factors linked to the specific manifestations of the condition can contribute to LDs being overlooked. In particular, when the condition is not severe, LDs can be managed up to secondary school, and it is reasonable to imagine that a number of students with LDs may reach higher levels of education without their LDs being detected [13,14]. At university, when task complexity increases, multitasking is often required (e.g., taking notes quickly and accurately, and listening to the lesson without losing information), and the volume of material to be processed increases. As a result, students might not be able to compensate for their difficulties any longer. In addition, young adults face an environmental demand to be more autonomous in studying and managing their academic career. All these factors connected to individual development and environmental changes may contribute to the emergence of difficulties that were not previously evident and might impact students’ learning proficiency, self-esteem, and self-efficacy [15,16]. The observed changes in the pattern of difficulties and the late emergence of specific symptoms contribute to increasing the complexity connected to performing a *late diagnostic evaluation.* Specifically, regarding the ability of reading and writing, it has been observed how crucial aspects such as difficulties in decoding single words and in applying the rules for a correct grapheme-phoneme conversion tend to decrease through different developmental stages [17]. Conversely, other difficulties, such as slowness in reading and writing non-existing words, specific terminology, foreign words and, in general, a documented deficit in applying lexical procedures, seem to remain stable over time [17,18]. Accuracy in decoding is generally less compromised and tends to improve over time, possibly reaching a performance level close to that observed in typical development [19]. Furthermore, the decoding speed plays a very important role in determining, aside from comprehension, performance in all school subjects, even in typically developing students [20]. 

As a whole, these characteristics may influence the presence of orthographic errors in the transcription of complex words, which usually require the direct retrieval of the orthographic representation to be accurately written. It has been reported that secondary school dyslexic students showed a significant *text-specificity* effect that translated into further difficulties whenever these students needed to handle more complex texts, in terms of morphosyntactic components, and with a high number of non-frequent words [21]. In general, the existing literature on the characteristics of dyslexia later in development outlines a complexity that is also connected to the fact that LDs often co-occur, in particular, dyslexia and dysorthography. Dyslexic and dysorthographic students experience a specific difficulty in automating the orthographic component when the task complexity increases, when a task is new and has never been performed before, or when difficulty and novelty are combined in a task. In this regard, [22] investigated which task would be the most effective in challenging dyslexic university students with respect to typical controls and documented how, in transparent languages, measures of phonological automaticity are the best indexes of reading and decoding competence, particularly in adults. The literature investigating which characteristics remain stable over time in a transparent orthography system, such as that used in Italy, shows how the slowness in decoding represents one of the core issues that defines the profile of these students [1,22,23]. Slowness in writing, in fact, shows a sensible worsening across time and constitutes a parameter for distinguishing dyslexic students with and without dysorthography at higher levels of instruction. In contrast, in this specific population, accuracy in writing does not seem to diverge dramatically with respect to typical peers in standard writing tasks; however, the performance tends to drastically decrease in tasks that interfere with a lack of automation of the writing processes, such as the abovementioned articulatory suppression condition, or during a challenging condition such as an exam. This lack of automation of the writing process also implies a specific difficulty in switching between the lexical route, which allows an individual to retrieve the stored spelling of the target word from the orthographic lexicon, and the sublexical route, which supports an individual’s ability to transcribe unfamiliar words [24,25,26].

Due to these characteristics, diagnostic tools must take into account the compensation strategies that students have established over time, and it is necessary to include specific diagnostic assessment tasks that are able to interfere with these compensation strategies (e.g., writing tasks to be executed in articulatory suppression). One important aspect of LDs in adulthood is assessment and diagnosis. Indeed, the majority of assessment batteries and tests are standardized for children, and very few instruments have been adapted and normed for adults. Furthermore, some dyslexic adults who had problems during their school years or were late in learning to read may have developed strategies for decoding with the support of comprehension, so that in adulthood, they may not display obvious literacy difficulties [27,28], but still present difficulties in complex and prolonged reading and writing tasks [29]. For this reason, specific tasks, such as dictation under a suppression condition, exhibited a very high sensitivity in discriminating between groups, even at a higher level than the one indicated as necessary by the literature [22,30,31]. This suggests that this procedure could be effectively used in the routine assessment of dyslexic and dysorthographic university students, as it might interfere with the strategies for compensation that adults and young adults have established over time.

### 1.2. Self-Efficacy Perception and the Impact on Career Decision Making and the Drop-Out Rate

“Self-efficacy” refers to “beliefs in one’s capabilities to organize and execute the courses of action required to produce given attainments” [32] (p. 3). Positive academic self-efficacy beliefs are associated with higher levels of motivation, higher levels of persistence, and overall academic success. There is a gap in the literature regarding the way in which young adults with learning disabilities who are enrolled in postsecondary education develop their academic self-efficacy beliefs and corresponding adaptive coping skills. Reed et al. [31] explained how students with learning disabilities in college display lower levels of self-efficacy beliefs than their non-LD peers. On the basis of their results, the researchers highlighted that students with learning disabilities in higher education reported less confidence in their capabilities to meet academic demands, questioned their overall academic competencies, and demonstrated increased pessimistic attitudes towards completing higher education requirements. Researchers have argued that lower levels of academic self-efficacy beliefs translate into a diminished sense of capacity for learning in challenging academic curricula [10]. Therefore, it may be argued that individuals with identified LDs are significantly more likely to encounter challenges with performance and motivation due, at least in part, to lower levels of self-efficacy beliefs. In contrast to peers with learning disabilities who express lower levels of self-efficacy beliefs, individuals with LDs who have positive and accurate self-efficacy beliefs are more likely to achieve independence and autonomy within postsecondary learning environments [11]. In the same vein, Wright et al. [33] found that a positive relationship involving positive academic self-efficacy, continued enrolment, and academic achievement levels existed for college students with LDs. The research results indicated that the reported levels of self-efficacy at the end of the first semester of college were related to academic success [33]. Understanding these characteristics in university students is of pivotal importance, not only to allow these students to progress in their learning and career path while lowering their risk of dropping out, but also to possibly identify students with a previously undiagnosed LD. As mentioned previously, undiagnosed students have to compensate for their difficulties to succeed, with a consuming active effort that is often not in line with their performance, and they are likely to internalize a sense of frustration and inefficacy. These feelings may impact dimensions such as self-esteem, self-image, and the general perception of self-efficacy, which, in turn, may impact future and long-term decisions, such as their choice of academic path, career, and job [34]. The results from interviews, self-reports, and tests with university students have shown that students with dyslexia have problems with a number of common academic tasks, e.g., note taking and expressing ideas in writing. Many of the students reported that their difficulties were long-standing and had already been experienced in elementary school and later during higher education [7,35]. Difficulties have also been reported to change over time [35], in line with the developmental trajectory that LDs exhibit throughout an individual’s life span [2].

### 1.3. The Present Study

The data used in this study were collected during a broad data collection effort performed in seven Italian regions with the participation of seven universities and aimed to standardize a new battery for assessing LDs in adults and young adults. The project was promoted by the University of Padua and coordinated by the curators of the battery. The authors of the present contribution participated in the standardization and collected data at the University of Florence and Genoa. 

In the Italian context, the evolution of norms relative to LDs and the enactment of law number 170/2010 [36], which recognizes dyslexia, dysorthography, and dyscalculia as LDs, have determined a growing interest in increasing the possibility of individualizing the learning context and supporting students with LDs with the use of technologies and other supportive tools, in order to preserve the learning experience of these students not only in early stages, but also later in development, and to extend the use of supportive tools and technologies for college and university students (law number 170/2010, art. 5, subparagraph 4). In addition, the guidelines with regards to the Ministerial Decree of 12 July 2011 clearly express that universities must take the lead in actively promoting solutions and tools in both teaching and assessment to foster proficient learning processes for older students with LDs. At the same time, there is an evident need for new diagnostic instruments adapted for this specific age range that would be able to offer a precise diagnostic process and access to supportive tools for adults and young adults that have not been diagnosed in the past, but who have experienced difficulties in learning. As mentioned, a prolonged experience of learning difficulties may lead to a lower self-efficacy perception and an increased tendency to drop out of colleges and universities.

From this perspective, the aim of this study was two-fold: (1) To compare the performance of inaccurate writers who were not diagnosed with LDs with that of students who were diagnosed with LDs, in order to identify which tasks are the most challenging for individuals with LDs, and (2) to investigate whether inaccurate writers with and without a diagnosis differ in terms of self-perceived difficulties.

## 2. Materials and Methods

### 2.1. Participants

Two groups were extracted from a sample of 639 university students (380 females, mean age 22.15 years, *Standard Deviation (SD)* = 2.18) attending seven Italian universities in both scientific and humanities programmes: 48 participants (24 females) who registered scores on writing tasks below the 5th percentile and 51 participants (24 females) who were diagnosed with LDs (dyslexia, dysgraphia, and dysorthography) in the years prior to the administration of the battery and who were included as a control sample in the data collection. The students’ participation in the study was voluntary, and recruitment was organized at the university prior to the study being presented to the students in classes with the help of the professors in charge of the courses. Informed written consent was obtained from participants before data collection began (they were all older than 18). All of the students included in the LD sample were diagnosed with dyslexia, dysgraphia, and dysorthography.

### 2.2. Procedures

The tasks selected for the present study were extracted from a newly standardized battery [37]. The complete battery was administered during two test sessions (mean durations of 60 and 40 min) in a quiet, dedicated room or laboratory at the hosting universities. During the first session, reading, writing, and calculation tasks were administered, whereas in the second session, which could be conducted in small groups, with students working independently or individually, a task of text comprehension was administered. The interval between the two sessions ranged from 1 to 2 weeks. Globally, data were collected in a six-month time frame. For the purpose of this study, we exclusively selected and included the writing tasks in the analysis. 

### 2.3. Description of the Selected Tasks

#### 2.3.1. Writing Tasks

##### Word Dictation Task

In this task, the experimenter dictated eight lists of words in two different conditions: Participants were asked to write down four lists in a normal condition (NC) and four in an articulatory suppression condition (ASC). Each list comprised 14 words that varied in length (long and short) and frequency (high and low). The high-frequency short words (HFS), e.g., “bosco” (“woods”), and low-frequency short words (LFS), e.g., “fiele” (“bile”), were composed of two syllables and comprised a total of 75 graphemes and 28 syllables per condition. The high-frequency long words (HFLs), e.g., “avvocato” (“lawyer”), and low-frequency long words (LFLs), e.g., “pianeggiante” (“flat”), were composed of four or five syllables and comprised a total of 144 graphemes and 61 syllables per condition.

Both conditions assessed the orthographic component, specifically the dictation task in the ASC, which allowed the assessment of not only the automation of the writing process, but also the student’s ability to maintain the orthographic characteristics of words in an interference condition.

##### Normal Condition Administration

In the normal condition, the experimenter dictated each list at a constant rate, typically 2 s per word, but maintained the flexibility needed to address any possible difficulties of the students.

Each error (e.g., each misspelled, omitted, or incomplete words) made by the student was scored as 1. Multiple errors in the same word were scored as 1. The response variable was the total number of errors made across the four lists.

##### Articulatory Suppression Condition Administration

In this condition, the student was asked to repeat the syllable *LA* out loud continuously while performing the dictation task. In this condition, the dictation rate was required to be constant, typically 3 s, but the dictation was allowed to be interrupted if the student temporarily suspended the articulation. If the student showed specific difficulties in following the rhythm of the dictation, the task could be paused and restarted at a constant rate. The response variable was the total number of errors made, scored as described above, across the four lists.

##### Text Dictation Task

The dictation of the text was meant to objectively assess the orthographic component of writing. In this task, the experimenter dictated a text out loud, carefully modulating the rhythm based on the students’ rapidity in writing. It is not possible to give explanations about phrases or words that may have been difficult to understand in any phase of the task administration. The student was required to base their writing solely on the context. Prior to the start of the administration, students were informed that they needed to pay attention to the words, as they could not be repeated, and if they fell behind in writing, they should skip the word and proceed with the task. The response variable was the number of errors, scored as described above.

##### Writing Numbers in Words Task

This is a classical task relating to the writing speed. The student was required to write down in words, using the preferred graphic font, as many numbers as possible within one minute starting from the number one. This task was also administered in both normal and articulatory suppression conditions. 

#### 2.3.2. Vinegrad+ (Adaptation of the Vinegrad Questionnaire)

The Vinegrad+ [38] is a self-report measure that presents a series of items aimed at investigating the perception of difficulties in everyday life tasks that require the automation of reading and writing processes and associated abilities, also considering the evolution of the documented difficulties of individuals with LDs transitioning into adulthood. It comprises 26 dichotomous items (“yes” and “no” responses), and the total score is the number of positive answers. The items can also be clustered, depending on the area of difficulty they are meant to investigate, thus making it possible to identify specific areas in which the student perceives they have the most severe difficulties. The four areas are (i) the general characteristics of LD, which comprises eight items that investigate the difficulties in everyday tasks that imply reading, writing, and related issues that are possible to automate with respect to social abilities and linguistic components; (ii) the reading score, which comprises six items that assess perceived difficulties in the orthographic and motor-graphic components (speed, accuracy, and comprehension) of reading; (iii) the writing score, which comprises six items that evaluate the orthographic components of writing; and (iv) the calculation score, which comprises six items that tap into the automation of arithmetic facts, mental calculation difficulties, and knowledge of writing calculation task procedures.

### 2.4. Statistical Analysis

Descriptive statistics (i.e., means, standard deviations, possible score ranges, skewness, and kurtosis), and zero-order and partial (Pearson) correlations among the measures were calculated (Table 1 and Table 2). 

To investigate which differences may exist in terms of the number of errors and speed in subgroups 1 and 2 (see the descriptive statistics reported in Table 2), after testing the association pattern between the variables, groups were compared using independent sample *t*-tests to investigate which tasks were more challenging for the LD group. We then used propensity score analysis (PSA [39]) to investigate the net group differences in self-perceived difficulties when matched with the writing tasks performance. PSA has been recommended as a more principled method than analysis of covariance to account for the imbalance of groups regarding relevant covariates, as the latter method is likely to provide biased and inconsistent estimates of group differences if not all relevant interactions and nonlinear effects are included in the model [40]. PSA was performed with the *matchit* function in the *MatchIt* [41] package in R using the *genetic* method [42].

## 3. Results

### 3.1. Descriptive Statistics and Correlations

Descriptive statistics and correlations are reported in Table 1 and Table 2. The writing tasks showed a pattern of significant correlations that supported the association among writing tasks, consistent with the pattern reported in the manual of the battery [37]. Significant correlations were also found within self-report measures and between writing tasks and self-report measures (see Table 1).

### 3.2. Independent Sample t-Test Results

The results of the independent sample *t*-tests showed that there were large differences (Cohen’s *d* > 0.80) in self-perceived difficulties between the 5th percentile and the LD group, but this result could be an artefact of small-to-large differences in writing task scores (Table 2). The two groups significantly differed in the articulatory suppression condition tasks, both in terms of the number of graphemes (a variable that measures slowness vs. fastness in writing) and accuracy (number of errors committed in both the word dictation task and the writing-numbers-in-words task), but not in the standard condition tasks, with the only exception of the dictation task.

### 3.3. Propensity Score Analysis

When we performed PSA to test net group differences in self-perceived difficulties, we found a large difference in pre-matching distance scores (i.e., the logit-transformed probability of a case of belonging to its actual group given the covariates—in this case, writing tasks scores) (LD group: 0.39 ± 0.23; 5th percentile group: 0.59 ± 0.17; *t*(87.85) = 4.96, *p* < 0.001, *d* = 0.99 [0.56; 1.41]). 

### 3.4. Matching Procedure Results

The matching procedure discarded 31 observations in the LD group that could not contribute to achieving a balance between the groups for the writing task scores. Hence, the final analyses were performed for the original 5th percentile group participants (*n* = 48), who were considered the reference group and thus assigned a weight of 1 by the procedure, and 21 participants from the LD group, who were considered the focal group and were assigned a weight ranging from 0.42 to 3.33 (median = 0.62) to reach an adequate balance. After matching, the difference in distance scores was no longer significant (LD group: 0.55 ± 0.16; 5th percentile group: 0.59 ± 0.17; *t*(67) = 0.20, *p* = 0.845, *d* = 0.05 [−0.46; 0.57]), and the groups were adequately balanced in terms of the writing task scores (Table 3). Nevertheless, the first three rows of Table 3 show that even after matching, the group differences in the self-perceived difficulties were still large, suggesting that they could not be accounted for by differences in the writing task performance.

## 4. Discussion

This study compared the performance of inaccurate writers who were not diagnosed with LDs with that of students who were diagnosed with LDs in an attempt to identify which tasks are the most challenging for individuals with LDs, while also investigating whether inaccurate writers with and without a diagnosis differ in terms of self-perceived difficulties. An innovative contribution of the present study is that it analysed the difficulties experienced by individuals with LDs that may last until young adulthood using a sample of inaccurate writers, performing below the 5th percentile in the writing tasks, as a control group. Moreover, we studied the impact that being diagnosed with LDs may have on the self-perception of difficulty, particularly with regard to writing skills. The results regarding writing tasks showed that the two groups significantly differed in the articulatory suppression condition tasks, but not in the standard condition tasks, with the only exception of the text dictation task. Furthermore, with regard to the perception of difficulty, students who were diagnosed with LDs reported significantly more perceived difficulties, pertaining to the writing tasks selected for the purpose of this research, than inaccurate peers. Students with LDs also reported significantly more perceived difficulties after the two groups were matched for performance on writing tasks.

The correlation analyses confirmed a homogenous pattern of association among writing tasks and between writing tasks and perceived difficulties assessed by the general and writing-related items and by the total score of the Vinegrad [44]. Consistent with the literature, the comparisons of the two groups only outlined significant differences in the most challenging tasks, namely, all the tasks administered in the articulatory suppression condition and, to a lesser extent, in the text dictation task in a normal condition. Although university students with LDs are presumably able to compensate for most of their difficulties when reaching higher education and their performance in many tasks can be comparable to that of students belonging to the extreme of the normal distribution, specific characteristics of individuals with LDs emerge when they face tasks that involve a higher cognitive load. In simple tasks such as word dictation and grapheme writing, their performance does not differ from that of inaccurate writers, whereas they encounter particular problems achieving this level of performance in more difficult tasks. The condition of articulatory suppression interferes with the activation of the word subvocal rehearsal strategy during transcription, which usually allows continuous retrieval of the composition of the word. Generally, students diagnosed with dyslexia and dysorthographia compensate by articulating words during transcription, and whenever they are unable to apply this strategy, their performance tends to decline. An inaccurate writer performance, conversely, seems not to be particularly challenged by this condition, in terms of mean scores, possibly because, globally, they managed to establish better access to coding. The rehearsal process implied in the articulatory suppression condition seems to interfere with the normal functioning of working memory: Students who are able to fully automate the writing process and orthographic rules and rely on the lexical representation of the words are not particularly affected by this interference effect. Literature reporting results on adults with dyslexia, on the contrary, documents that dyslexic adults are strongly influenced by this specific interference effect [45]. From this perspective, students with LDs may need to rely on the sublexical route (accessed via subvocal rehearsal) not having immediate access to intact phonetic representations [17], which would also support a rapid retrieval of word representations while writing. Overall, considering the same tasks when performed under normal conditions, our results show that students who were diagnosed with LDs did not differ with respect to inaccurate writers.

The text dictation task, in which the two groups showed significant differences, represents an exception to this trend in terms of accuracy. This result may be due to the difficulties that writing under dictation for a more prolonged interval of time may present, in terms of the rhythm that needs to be followed and time, which may not allow the use of the subvocal rehearsal, especially when words are long, infrequent, and dictated continuously. Moreover, during the text dictation task, the students need to follow the experimenter’s dictation rhythm by writing at the same pace, and the perceived need to be fast (although the experimenter was instructed to adapt, to some extent, to the rhythm of the participant) might interfere with the LDs participants’ performance and result in a more inaccurate outcome. Interestingly, when considering standard tasks, such as word dictation and grapheme production, students with LDs did not seem to make more errors than students who were within the tails of the normal distribution. The tasks performed under the articulatory suppression condition cannot be classified as standard everyday tasks that were familiar to the student, but were specifically created to discriminate the specific characteristics of LDs [22]. 

The differences between the two groups registered in the Vinegrad+ questionnaire are particularly interesting. This self-report measure specifically focuses on the perception of difficulty, and the results clearly indicate that participants who were already diagnosed with LDs tended to report significantly more perceived difficulties compared to inaccurate writers who had never been evaluated for or diagnosed with LDs.

The analysis performed on the matched samples allowed us to test whether the differences found between the perceptions of the two groups were linked to the actual differences found in the performance of specific tasks that we described above. The results confirmed that even when balancing the performance of the two groups for all tasks under the articulatory suppression condition, which emerged as the most discriminative for students with a diagnosis of LDs, participants with LDs reported significantly more perceived difficulties than students identified as inaccurate writers—who, in some cases, possibly had an LD that had not yet been detected. This result, in particular, may be of specific interest in discussing the all-around implications of being diagnosed with LDs and the need to work not only on the learning cognitive mechanism, but also on more general aspects, such as self-esteem, self-efficacy, and self-worth. The literature indicates how students with LDs benefit more from specific activities, such as first-year preparation courses, before entering university [31], as they show greater improvement in terms of academic self-efficacy compared to their typical peers. In general, these results highlight the importance of analysing all the emotional aspects that may be connected to and triggered by a diagnosis of an LD in depth. Being diagnosed with an LD can increase students’ awareness of their own difficulties, with possible positive effects on the use of effective learning and metacognitive strategies [46,47]. Nevertheless, it is important not to underestimate the possible negative impact of the diagnosis on self-efficacy and self-esteem, which must be taken into account when guiding students at any level of schooling.

The emerged enhanced perception of difficulty is a finding consistent with the limited existing literature on the topic. The results from a comprehensive study on the efficacy of university preparation courses by Reed et al. [31] outlined that students with learning disabilities in college report lower levels of self-efficacy beliefs than their non-LD peers. Reed et al. [31] highlighted that students with learning disabilities in higher education tend to report less confidence in their capabilities to meet academic demands, question their overall academic competencies, and show increased pessimistic attitudes towards completing higher education requirements. Lower levels of academic self-efficacy beliefs are argued by researchers to translate into a diminished sense of capacity for learning challenging academic curricula [10]. Consistent with the limited existing literature on the topic, these results support the idea that individuals with identified LDs are significantly more likely to encounter challenges in maintaining a good level of motivation and persistence in trying to overcome the difficulties they face, on a daily basis, due to lower levels of self-efficacy beliefs. In the long run, unlike peers with LDs who express lower levels of self-efficacy beliefs, individuals with LDs who have positive and accurate self-efficacy beliefs are more likely to achieve independence and autonomy within postsecondary learning environments [11].

The results presented in the current study show that the enhanced perception of difficulty concerns not only specific difficulties related to writing skills, but also general learning features (considering everyday activities that imply reading and writing processes, e.g., consulting a map, and one’s abilities relative to linguistic components that can be automated) and the overall learning experience assessed by the Vinegrad+ total score. These results suggest important areas for intervention that not only take into account the cognitive features and challenges that individuals with LDs face, but also the emotional impact of a diagnosis on the general perception of ability that, in turn, may influence future outcomes, such as career choice and the drop-out rate in higher levels of education [48]. This attitude towards education and learning, in fact, may negatively influence one’s career path choice, directing individuals towards something that they perceive as easier and/or less demanding, instead of something that is truly of interest to them and that they feel passionate about, with a subsequent impact on their future work life and, possibly, an enduring sense of general frustration and low-self efficacy beliefs. From this perspective, working on this emotional facet of the condition would not only magnify the effectiveness of the cognitive training on the implementation of strategies, but would also result in a protective factor that could reasonably reduce feelings, such as anxiety, and a perceived lack of efficacy, increasing the ability to tolerate fatigue and frustration; working on this facet of the condition would also preclude the creation of negative emotional anticipation associated with learning and education.

In conclusion, the results of the present study are consistent with previous findings on the compensation that university students with LDs are able to implement, on one hand, and on the tendency of these students to internalize an experience of difficulty and fatigue with regard to learning, on the other hand [30,31].

Some limitations of this study warrant mentioning. First, as students with LDs are relatively rare in Italian universities, the sample size was inevitably not very large, and this might have impacted the statistical power. Future research in this domain should attempt to replicate these results using larger samples. Second, we only used one self-report measure for perceived difficulties: The use of multiple measures and the collection of data from observers, such as peers, teachers, tutors, and/or parents, will help to provide a more comprehensive assessment of the phenomenon. Despite these limitations, this study adds knowledge to a relatively less investigated stage of development and the features of LDs in young adulthood. In addition, it provides evidence of differences in the individuals’ perception of difficulties while controlling for performance, suggesting important areas for intervention. From this perspective, these results address the crucial role that receiving a diagnosis has in guaranteeing that students with LDs have access to all the instruments and tutelage that can allow them to proficiently experience learning at university. Moreover, the results provided by the present study highlight the importance of investing in practices that support students emotionally, with a specific focus on self-efficacy and self-esteem beliefs, which, together with the above mentioned tutelages and instruments, constitute a core protective factor for preventing drop-out, allowing students to confidently persevere in their careers. An important advancement in this regard would be the implementation of a specific support service at university to work with LD students on both the emotional impact of diagnosis and specific strategies and methodologies for supporting them in studying. These practices would increase and support student’s self-efficacy and self-esteem, working on both performance and academic “identity” at the same time.

## 5. Conclusions

Overall, in this study, we showed how students with LDs and writing difficulties can reach a certain level of efficient compensation. Nevertheless, students with LDs perceived significantly more difficulties with respect to their undiagnosed peers. This result was also confirmed when matching the two groups in terms of writing task scores, suggesting the importance of addressing self-perception and self-efficacy issues in these students.

## Figures and Tables

**Table 1:** Bivariate correlations between writing tasks and the Vinegrad+ questionnaire subscales.

	Text-Dictation	Words_NC	words_SC		Graphemes-		Graphemes-			
	Errors	Errors_	Errors	Graphemes-NC	Errors- NC	Graphemes-SC	Errors- SC	Vinegrad- Total	Vinegrad-GC	Vinegrad Writing
Text-Dictation-Errors	1.00									
Wors-Errors- NC	0.70 **	1.00								
Words-Errors-SC	0.72 **	0.64 **	1.00							
Graphemes-NC	0.05	0.00	0.11	1.00						
Graphemes-Errors-NC	0.33 **	0.23 *	0.26 *	0.42 **	1.00					
Graphemes-SC	−0.07	−0.03	−0.12	0.66 **	0.21 *	1.00				
Graphemes-Errors-SC	0.35 **	0.27 **	0.52 **	0.29 **	0.63 **	0.26 *	1.00			
Vinegrad-Total	0.22 *	0.10	0.42 **	−0.06	0.08	−0.26 *	0.21 *	1.00		
Vinegrad-GC	0.18	0.06	0.37 **	−0.07	0.01	−0.26 *	0.18	0.88 **	1.00	
Vinegrad-Writing	0.25 *	0.13	0.38 **	−0.07	0.16	−0.25 *	0.18	0.86 **	0.66 **	1.00

Note: **: *p* < 0.01; *: *p* < 0.05. NC = normal condition; SC = articulatory suppression condition; and Vinegrad_GC = Vinegrad general characteristics sub_scale.

**Table 2:** Prematching descriptive statistics and results of the independent sample *t*-test.

	Group		Levene’s Test for Equality of Variances		Independent Sample *t*-Test				
	5th Percentile (*n* = 48)	LD (*n* = 51)	F	*p*	*t*	df	*p*	adj-p	d
Text-Dictation-Errors	8.31 (4.52)	11.24 (8.01)	3.15	<0.001	2.25	79.79	0.027	0.039	0.50 [0.10; 0.90]
Words-Errors-NC	5.58 (4.33)	6.84 (6.52)	2.26	0.005	1.14	87.46	0.258	0.286	0.24 [−0.15; 0.64]
Words-Errors-SC	13.69 (8.11)	21.25 (12.89)	2.53	0.002	3.52	84.92	0.001	0.002	0.76 [0.35; 1.17]
Graphemes-NC	130.75 (38.48)	130.86 (35.31)	0.84	0.549	0.02	97.00	0.988	0.988	0.00 [−0.39; 0.40]
Graphemes-ErrorsNC	1.31 (1.65)	2.29 (3.35)	4.12	<0.001	1.86	73.88	0.066	0.083	0.43 [0.03; 0.83]
Graphemes-SC	113.46 (37.42)	95.12 (36.41)	0.95	0.848	−2.47	97.00	0.015	0.025	0.50 [0.10; 0.90]
Graphemes-ErrorsSC	2.00 (2.44)	3.52 (3.35)	1.88	0.031	2.59	91.42	0.011	0.023	0.54 [0.14; 0.94]
Vinegrad-Total	7.10 (4.24)	15.00 (5.41)	1.63	0.093	8.05	97.00	<0.001	<0.001	1.64 [1.18; 2.09]
Vinegrad-GC	1.98 (1.44)	4.06 (2.01)	1.97	0.021	5.95	90.50	<0.001	<0.001	1.25 [0.82; 1.68]
Vinegrad-Writing	1.73 (1.61)	3.78 (1.63)	1.03	0.932	6.31	97.00	<0.001	<0.001	1.28 [0.85; 1.71]

Note: df: degrees of freedom; *p*: raw *p*-value; adj-*p*: false-discovery-rate adjusted *p*-value [43]; and d: Cohen’s d, with its 95% confidence interval. NC = normal condition; SC = articulatory suppression condition; and Vinegrad_GC = Vinegrad general characteristics sub_scale.

**Table 3:** Postmatching descriptive statistics and results of the independent sample *t*-test.

	Group		Levene’s Test for Equality of Variances		Independent Sample *t*-Test				
	5th Percentile (*n* = 48)	LD (*n* = 20)	F	*p*	*t*	df	*p*	adj-p	d
Text-Dictation-Errors	8.31 (4.52)	8.45 (4.45)	1.02	0.458	0.45	66.00	0.809	0.991	0.12 [−0.40; 0.64]
Words-Errors-NC	5.58 (4.33)	6.60 (5.38)	1.62	0.089	0.77	66.00	0.668	0.991	0.21 [−0.32; 0.73]
Words-Errors-SC	13.69 (8.11)	15.09 (6.86)	0.75	0.212	0.02	66.00	0.991	0.991	0.01 [−0.52; 0.53]
Graphemes-NC	130.75 (38.48)	134.15 (25.83)	0.47	0.019	0.43	51.82	0.672	0.991	0.11 [−0.41; 0.64]
Graphemes-ErrorsNC	1.31 (1.65)	1.30 (1.36)	0.71	0.170	0.20	66.00	0.919	0.991	0.05 [−0.47; 0.57]
Graphemes-SC	113.46 (37.42)	110.25 (28.25)	0.60	0.079	0.06	66.00	0.976	0.991	0.02 [−0.51; 0.54]
Graphemes-ErrorsSC	2.00 (2.44)	2.30 (1.87)	0.61	0.089	0.03	66.00	0.986	0.991	0.01 [−0.51; 0.53]
Vinegrad-Total	7.10 (4.24)	14.20 (5.38)	1.70	0.071	6.71	66.00	<0.001	0.001	1.81 [1.20; 2.41]
Vinegrad-GC	1.98 (1.44)	3.85 (1.81)	1.67	0.077	5.29	66.00	0.001	0.006	1.43 [0.85; 2.00]
Vinegrad-Writing	1.73 (1.61)	3.45 (1.66)	1.13	0.358	5.06	66.00	0.002	0.006	1.37 [0.79; 1.93]

Note: df: degrees of freedom; *p*: raw *p*-value; adj-p: false-discovery-rate adjusted *p*-value [43]; and d: Cohen’s d, with its 95% confidence interval. NC = normal condition; SC = articulatory suppression condition; and Vinegrad_gen_char = Vinegrad general characteristics sub_scale.

## Data Availability

Data sharing not applicable. No new data were created or analyzed in this study. Data sharing is not applicable to this article.
